# Evaluation of video-assisted HPV education in government-supported clinics in Western Kenya

**DOI:** 10.1371/journal.pgph.0002539

**Published:** 2023-12-18

**Authors:** Haley Dion, Hanul Choi, Michelle Huang, Laya Sathyan, Emily Herfel, Breandan Makhulo, Jeniffer Ambaka, Saduma Ibrahim, Megan Huchko

**Affiliations:** 1 Center for Global Reproductive Health, Duke Global Health Institute, Durham, North Carolina, United States of America; 2 Department of Obstetrics and Gynecology, Duke University School of Medicine, Durham, North Carolina, United States of America; 3 Kenya Medical Research Institute, Nairobi, Kenya; University of Alabama at Birmingham, UNITED STATES

## Abstract

Despite prevalent preventative methods of human papillomavirus (HPV), cervical cancer remains the foremost cause of cancer-related death among women of reproductive age in Western Kenya. HPV self-sampling is a preventative measure that can improve accessibility and availability to cervical cancer screening. Correct education about HPV is crucial to combating stigma and increasing HPV screening uptake. In this study, we evaluated the workflow impact of a video-assisted HPV education to promote self-sampling in clinical settings in Kisumu, Kenya. We conducted a descriptive workflow study nested in a two-part cluster-randomized control trial in six government-supported health clinics in Kisumu County. We observed the workflow of HPV screening video-assisted and standard health educations. and evaluated community and clinic health assistant facilitation (CCHA), duration, and feasibility of the intervention. Thirty HPV screening-eligible women who participated in the video intervention were recruited for three focus group discussions (FGDs). The FGDs aimed to better understand women’s experience with the video screening, their impressions on the content, and feedback about intervention logistics. Across 33 observations, 16.5 women per day watched the educational video at intervention clinics, and 14 women per day heard standard Ministry of Health cervical cancer prevention education talks at control clinics. Sixty-three percent of women participated in HPV self-sampling in the intervention sites, compared to forty-six percent who screened after standard health talks at control sites. The workflow observations identified variable video projection and viewing space, access to power supply, and CCHA availability and ability to utilize the projector as major factors impacting education workflow. Women in FGDs appreciated the video modality, length of video, and education location. HPV video education is a suitable intervention, with further research recommended to determine the viability of sustainably implementing the intervention in a clinic environment. This research is fully funded by the Duke University Global Health Institute.

## Introduction

Cervical cancer is the leading cause of cancer-related death for women worldwide with about 90% of cases and deaths occurring in low- and middle- income countries like Kenya [1]. This disparity in the burden of disease remains despite the availability of vaccination and screening for human papillomavirus (HPV). These powerful preventive measures have inspired the WHO to call for a global strategy to achieve cervical cancer elimination in the next century [2]. Kenya, an East African country with high rates of cervical cancer, included HPV-based screening in the National Cancer Prevention Guidelines in 2018 and rolled out HPV vaccination in 2019 [3]. Despite governmental initiatives for community-based delivery of these preventive measures, screening and vaccination coverage is still very low, and there is a need for better public understanding of their availability and importance [4].

Many programs have implemented HPV testing via self-collection to address multiple concerns. It addresses the potential hesitancy among patients to seek screening, as well as the healthcare infrastructure and workforce needs related to exam-based screening such as visual inspection with acetic acid or cytology [5]. While removing the need for a pelvic exam, HPV self-sampling has potential to improve acceptability, accessibility, and availability of screening. HPV self-sampling can also facilitate a model of service delivery by community health volunteers or other lay health workers in provider limited areas. Although self-sampling availability is increasing, women in low-resource settings are still unaware of this option and the overall importance of screening. Prior research has found that women living in rural areas lacking formal education are much less likely to seek HPV screening than educated, high-income individuals [6]. The lack of awareness of HPV, its role in the development of cervical cancer, and personal perception of risk may negatively impact screening uptake. Educational messaging that effectively addresses the drivers of HPV-related stigma may work to increase both awareness and risk perception, increasing acceptability of screening [7].

Video-based educational interventions can foster health-seeking behaviors when offered alone or as an adjunct to other health messaging. A systematic review of 28 studies showed that video education interventions are effective in modifying health behaviors such as HIV testing and treatment adherence [8]. An educational intervention for women in Ghana that incorporated a variety of learning modalities, such as lectures from registered nurses, discussions, videos, and leaflets, resulted in increased knowledge of cervical cancer and HPV screening [9]. Researchers in Tanzania showed an increase in baseline knowledge on HPV infection and cervical cancer among women after watching a 15-minute HPV-focused video [10]. Videos have the potential to provide clear and standardized messaging, an important strategy to reduce misinformation in settings where health counseling is provided by lay health workers. Another study found that video-based reproductive health educational interventions are effective in increasing understanding of cervical cancer and HPV, preventive measures, and perceptions of these measures such as personal risk and screening stigma [11]. Use of videos featuring women who have gone through the screening cascade can facilitate peer-to-peer education, a known strategy for stigma prevention, in areas where experienced patients are not always available [12].

In a clinic setting, video-based education may play an important role in improving screening uptake through increased knowledge, risk perception, and reduced stigma toward HPV and cervical cancer. Challenges with implementation of a video-based intervention, especially in resource-limited clinics, include overwork of clinic assistants, technological barriers, and access to HPV self-sampling resources. To address the logistics and challenges of carrying out video-assisted interventions in low-resource settings, we sought to evaluate the feasibility of implementing a locally developed, context-specific video-based HPV education module as an adjunct to community and clinical health assistant (CCHA) health talks in government-supported clinics in Kisumu, Kenya. We carried out workflow observations and focus group discussions to explore how the educational video was implemented in a clinical setting and to identify facilitators, barriers and the possible impact of the intervention.

## Materials and methods

### Study design and intervention

**Fig 1:**
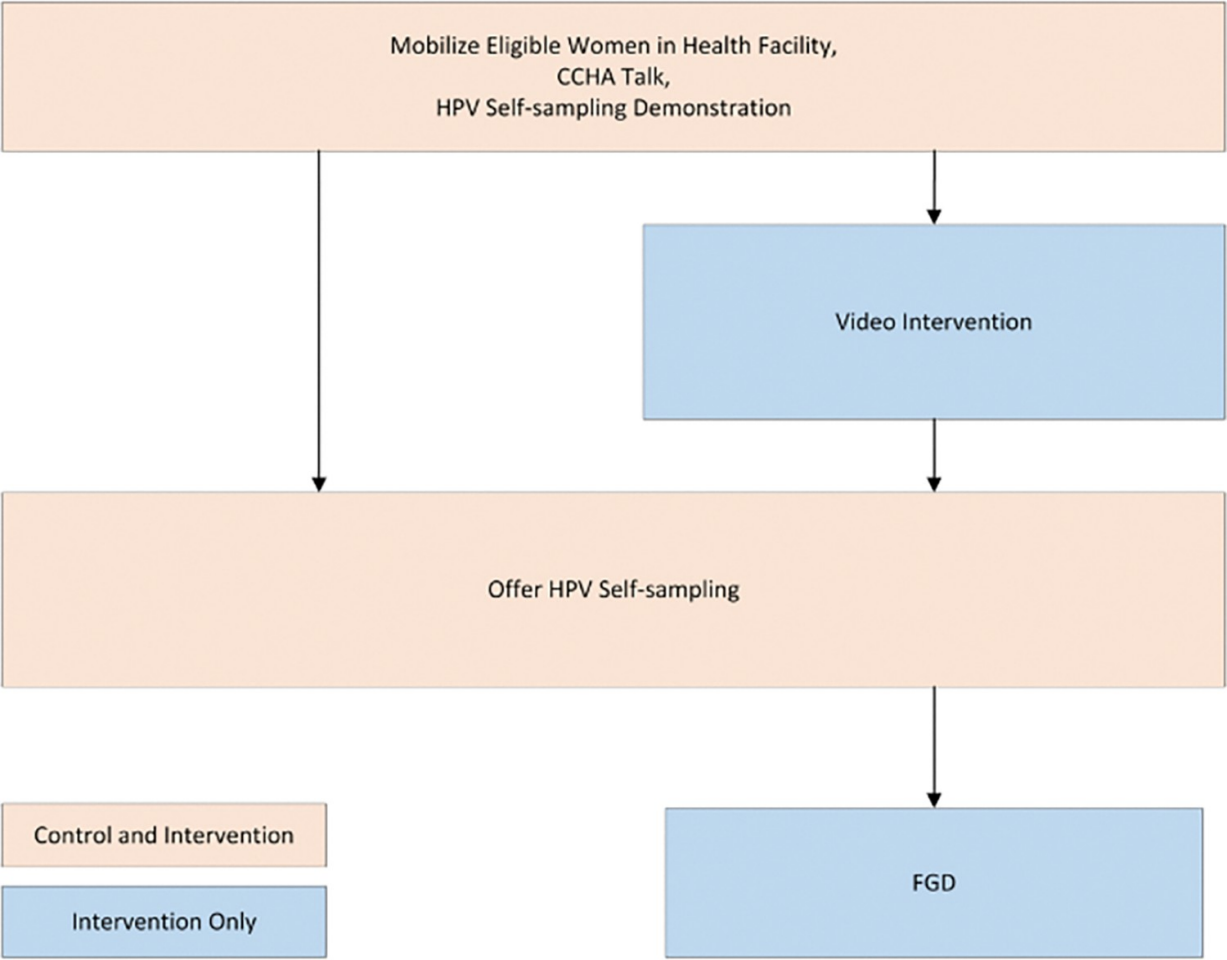
Overview of the study’s control and intervention arms, including both workflow and FGD procedures. The intervention clinics contain an additional step of video screening after the health talk and HPV self-sampling demonstration by the CCHAs. Women who watched the video at the intervention clinics were recruited for FGDs.

We conducted a two-part study to evaluate the feasibility and workflow impact of an HPV-focused educational video offered as an adjunct to in-person health talks. This study was nested within an ongoing cluster-randomized control trial in six government-supported health clinics in Kisumu County, Kenya. The parent trial sought to compare the overall impact of video-assisted health talks to standard educational health talks on screening uptake in the clinic setting. For the parent trial, six government-supported health clinics provided HPV self-sampling for women, free of charge. In the three control sites, CCHAs offered HPV self-testing with a standard educational talk to women attending the clinic, whereas three intervention clinics CCHAs offered educational talks enhanced with the video. The trial outcomes included differences in screening uptake between two arms. This paper describes an observational study to assess the workflow impact and user experience of the educational video intervention. Researchers carried out workflow observations at both control and intervention sites using structured tools. In order to better understand women’s experiences with the video and gain actionable feedback for future implementation, we carried out three focus group discussions (FGDs) among women who had attended intervention clinics (Fig 1).

The 5-minute HPV awareness and prevention video script was developed based on feedback from local sexual and reproductive health working groups and women in the screening target population. Using a peer-education model, the video consisted of a woman from the local community describing her experience with HPV self-sampling, result notification, and treatment for a positive HPV diagnosis, with a focus on social and emotional issues, including her feelings of fear, relief and empowerment. The video provided additional information about the screening process to supplement the description of HPV self-sampling in health talks provided by CCHAs. Videos were made in English and Dhluo with Kiswahili subtitles on both videos, reflecting the language preferences of the patient population. The video was created with culturally sensitive language, did not include explicit graphics or sensitive infographics, and went through several rounds of review and edits based on feedback. After delivering health talks and providing self-testing demonstrations, CCHAs answered any remaining questions prior to offering screening. Collected samples were logged into both a written logbook and an online database (REDCap, v12.4.28; Vanderbilt University 2022) using a QR code.

In this study, all CCHAs provided the standard Ministry of Health (MoH) cervical cancer prevention education at all six clinic sites, followed by an offer of HPV screening via self-collection. Health education topics included HPV infection process, standard HPV testing methods, and self-sampling. In the three intervention sites, the HPV educational talk was supplemented with the video, in which a woman discussed her experience with the HPV self-sampling, results notification, and treatment process. While both the control and intervention CCHAs were trained to give accurate health information using the same scripts about HPV, cervical cancer prevention and the self-sampling process, CCHAs at intervention sites received an additional half-day and on-site training in using stigma-reductive language and arranging projectors for the video play. CCHAs in both control and intervention arms either worked alone or in pairs to provide services.

### Workflow study methods

To evaluate workflow processes related to both the standard and video-assisted educational strategies, we sought to observe the process of HPV education and screening provision at least five times at each clinic. During each observation, researchers completed a questionnaire with both quantitative and qualitative measures, including factors such as the duration of each health education cycle, the number of women who participated in the health education, the number of women who opted into HPV self-sampling, the location that the video was shown in the intervention clinics, the number of women who participated in the education with or without dependents, potential interruptions (e.g., power outage), and other HPV-related services offered at clinics such as visual inspection after acetic acid application (VIA). Due to the novel nature of this intervention, we developed these instruments to assess the feasibility of the video based educational intervention and piloted the instruments within this study. Domains probed included length of respective education cycles, types of healthcare workforce engaging in education delivery, and potential factors for the disruption in intervention delivery. Two researchers were present during each observation day and separately filled out the survey questionnaires. CCHAs were instructed to collect the age of women interested in HPV self-sampling to ensure only screening eligible women participated (ages 30–64). The overview of survey instruments is included in Table 1, and a comprehensive table of survey instrument is listed in S1 Table.

**Table 1:** Overview of instruments for REDCap survey for control and intervention.

Instrument	Clinic Category	Description
Length of Education Cycle	Both	An average cycle duration of CCHA health talk, demonstration, and video play in intervention clinics to the nearest integer minute
Days Health Talks Provided	Both	Days that CCHAs delivered health talks (and video showing in intervention clinics) in previous week (Monday to Friday, multiple answer choice available)
HPV Self-Sampling Uptake	Both	Number of women receiving HPV self-sampling in each observation (only integer)
Video Interruptions and Types of Interruption	Intervention	Incidents when video screening was interrupted
Frequency of Video Screening	Intervention	Number of times that video was shown during the observation
Women with Dependents during Health Talks	Control	Number of women with dependents listening to CCHA’s health talks
Health Talk Delivery Interruptions and Types of Interruption	Control	Incidents when CCHA had to stop delivering health talks to patients

Study data was collected and managed using a REDCap database (v12.4.28; Vanderbilt University 2022) on handheld tablets. Descriptive instruments, such as additional comments, were analyzed by four team members using a qualitative coding method, and these were captured simultaneously with the quantitative data collection. All quantitative data analyses were performed using R Statistical Software (v4.1.2; R Core Team 2021).

### Focus group discussion methods

After the workflow study, FGDs were held at each intervention location. Guides were translated from English to Dhluo, and FGDs were carried out by facilitators who are native bilingual speakers. CCHAs used a screening questionnaire that assessed participants’ eligibility (ages between 30 and 65, video screening at intervention sites) and comfort speaking in front of a group. Participants were later contacted to return to the clinic for the FGD, at which time they provided written informed consent and were compensated for their transportation. Each FGD, consistent of 9 to 10 participants, took two to three hours.

The FGDs started with a group viewing of the video after which participants were asked questions about their experience with the video and their impressions on the content and its potential effectiveness as an educational tool. Data from the FGDs was used to supplement the workflow observations to better understand barriers and facilitators to implementation of the video in the clinic setting and women’s experience of watching the video in a group setting.

The audio recordings of the FGDs were transcribed in Dhluo, then translated into English and reviewed for accuracy by the facilitators. The initial codebook was developed prior to the FGD, and a rapid thematic analysis was conducted to provide qualitative feedback, prepare for the second review, and identify potential themes missing in the initial codebook. Then, the team came to an agreement to include potential themes to qualitative coding process. Four team members (HC, HD, MH, LS) conducted qualitative coding using NVivo (v12; QSR International 2021)

### IRB approval

The study received IRB approval from both Duke University Health System (Protocol ID: Pro00101931) and the Kenya Medical Research Institute (KEMRI) (KEMRI/RES/7/3/1).

### Inclusivity in global research

Additional information regarding the ethical, cultural, and scientific considerations specific to inclusivity in global research is included in the Supporting Information (S1 Text).

## Results

Between June and July 2022, we carried out 33 observations in the six clinics, 17 in the three intervention and 16 in the three control clinics. An average of 16.5 women per day saw the educational video after HPV health talks at intervention clinics, and 14 women per day heard HPV health talks at control clinics. The video was shown an average of 3.1 times per day at intervention clinics. For one clinic that employed both group and individual health talks, the data may not be unique to women in their first cycle of education for the total number of women educated per observation or the total number of health talks. At intervention clinics, each health education and video cycle lasted for an average of 17.6 minutes (video content at 5 minutes), whereas each standard health education cycle lasted 8.4 minutes at control clinics. Throughout the course of the day, the HPV education, excluding time to setup equipment, took 54.8 minutes at intervention sites, whereas standard health talks took 20.5 minutes over the course of an observation. Set up times varied across the intervention clinics but took up to 45 minutes.

At intervention sites, among the 307 screen-eligible women who saw the video-assisted health talk, 182 (63%) opted into HPV self-sampling after CCHAs delivered health education, compared to the 103 out of 224 (46%) women who screened after standard health talks at control sites. Among the 57 women attending clinics with dependents who watched the video, 37 (64.9%) received HPV self-sampling at intervention clinics, whereas 26 out of 129 (20.2%) women with dependents who listened to the health talk followed-up with screening at control clinics. Women who participated in the education at intervention clinics showed more signs of engagement with the information, such as asking more questions and participating in discussion with the CCHAs and their peers, as compared to women in the control sites.

The workflow observations identified factors impacting the ability to consistently offer the educational video screening, including variable projection and viewing space, access to power supply, and availability of a CCHA to understand how to play the video. The video was intended as an adjunct to the health talks and was not observed as a replacement for CCHA-led health talks. Technological difficulties with the video were observed despite brief trainings and on-site assistance in troubleshooting, and CCHAs sought assistance from research staff when challenges took place during the intervention. Difficulties that occurred include switching video language settings between English and Dhluo and using new screening methods, such as switching from playing the video on a projector to a laptop due to a power outage. Researchers observed technological challenges in 5 out of 17 observations days, with only 2 causing interruptions to the video education (i.e., power outages). In both instances, the video was able to be shown after resolution of challenges. There were no observations when the video was not shown.

Availability of and task sharing strategies among CCHAs played a significant role in the consistency of video-education and screening at the clinics. Four of the six clinics had two CCHAs who worked together to provide education and conduct screening, while the other two clinics, one in the intervention and one in the control arm, only had one CCHA for the entirety of the study. For clinics with one CCHA, the CCHA had to independently manage the education, screening, and data input. Researchers noted less health talks delivered to complement video screenings and a lower number of women screened after health talks, in comparison to the intervention clinics with two CCHAs. On average, the clinic with one designated CCHA would play the video 3.5 times per observation compared to 4.25 times in a clinic with two CCHAs. Researchers observed that among clinics with CCHA pairs, CCHAs collaborated to help ensure video education and online data collection could occur simultaneously with screening administration. When in pairs, CCHAs divided roles (screening administration and education) according to preference or expertise (previous experience with the online data collection method). In the clinics with CCHA pairs, there were four observed days when one of the CCHAs was absent, during which the remaining CCHA experienced challenges in handling their partner’s typical responsibilities and received assistance from research staff. An instance was witnessed by the research team, in which one CCHA required encouragement from researchers to conduct the video education after their partner, who typically provided the education, was absent. In that instance, the research coordinator aided in the delivery of video education to ensure that education was provided to women prior to screening. CCHAs played the video on average 3.5 times per observation when their partner was absent, similar to averages in clinics where one CCHA was the norm. Overall, researchers found unique strategies among CCHAs that presented benefits to help inform the implementation of larger scale projects where an increased number of women would be educated and screened.

During focus group discussions, participants identified interrupting factors from the clinic as potential distractions to the video watching experience. Participants brought up difficulty in looking at the visual components, such as the subtitles, due to bright lighting and wrinkled projector screens in the clinics, though they were still able to understand the subtitles. Some were distracted by crying children and many mentioned that they were called to see a clinician during the education, which interrupted their viewing of the entire video. Despite these interruptions, women mentioned they were able to watch the entire video because it could be replayed by the CCHAs when they finished their appointment.

While participants found the current length of the video acceptable, they also suggested that it could be lengthened to include more information regarding screening during menstruation and pregnancy and to clearly explain acronyms such as HPV and VIA. They expressed appreciation for the language options of both Luo and English and comfort with the type of language used in the video, describing it as appropriate, clear, and non-stigmatizing. Participants found the group setting appropriate to watch the video, and they expressed their comfort watching the video alongside their acquaintances, male clients, and children due to its inoffensive language use and relevance within the population. They reported a preference for watching the video in a group setting due to increased engagement in discussing the content with other women at the clinic, citing peer support during health education as a reason for screening. Participants also reported feeling encouraged to ask HPV-related questions to CCHAs when among peers. Women expressed the potential benefit of offering the video beyond the clinic setting, such as allowing it to be shown in schools, churches, and markets to reach a wider audience with education.

Most women expressed their preference in receiving health education through a video rather than written materials, suggesting that video education modality increases education access to those who may be illiterate or may not have time to read written materials at the clinic. They expressed a desire for having a one-on-one talk with an educator in conjunction with the video so that any remaining questions could be answered. Participants enjoyed the health talks the CCHAs gave prior to the video and stated that the CCHAs’ emphasis on HPV as a treatable condition made them feel more comfortable and increased their likelihood to screen. Despite the positive aspects of the CCHA health education talk before the video intervention, women’s trust in the CCHA’s varied, with some women pointing to them as a potential source of misinformation due to incorrect knowledge of screening eligibility criteria. However, some expressed seeing the video to check the validity of CCHA’s claims and were motivated to watch the video after listening to the CCHAs talk.

## Discussion

We found that implementing a video-augmented health education intervention to increase HPV knowledge and to promote HPV self-screening in government supported clinics in Western Kenya was feasible and acceptable. The video was shown consistently as intended by CCHAs across intervention clinic locations as an adjunct to their health talks and was not seen to replace the live talks. Women expressed approval of video as an educational modality, and appreciated the education’s content, language usage and social setting. More women participated in HPV-related cervical cancer education and had a higher likelihood of screening in intervention sites compared to women in control sites, suggesting the impact of video enhanced cervical cancer prevention education. We identified barriers such as CCHA’s familiarity with technology use and their capacity to assist clients for HPV screening that may affect the sustainability of this intervention.

Our finding that women in FGDs preferred video-based education reflects prior research of the impact of visual learning to convey behaviors and concepts to viewers that are harder to depict solely in words, and it increases access to information by those with a lower literacy level or those who did not have the time to read written materials [13]. The video reinforced knowledge about HPV and served to clarify potential misinformation from CCHA-led health talks. Women expressed their appreciation for the content and use of clear, accessible, and easily understandable language in the video, which made it more acceptable to watch in the clinic setting and potentially disseminated more widely for broader reach. The length of the video was also found to be acceptable, but it could potentially be expanded to include additional information to increase women’s knowledge.

Watching the video as a group motivated women to stay for the entire education and participate in the intervention more actively, as seen through engagement in discussions and questions. Women mentioned that peer influence while watching the video encouraged them to screen, suggesting positive impact of discussions within the group health education. This is in concordance with Albert et al. (2007)’s study showing how viewing educational videos at home with family members or within a group setting is beneficial to modify risky behaviors on heart failure [14]. Women were also comfortable watching the video with men and children, and recommended that the HPV educational video be disseminated beyond clinical settings, such as virtual spaces (online), schools, churches, and markets to attract more viewers and reach a wider audience. This suggests the potential target audience of the video-based educational intervention could expand beyond reproductive aged women to potentially increase social support among partners and diminish stigma when watching the video with others.

Our analysis also identified specific points of improvement to make the video intervention more sustainable in the clinic setting. The observed environmental distractions and lack of CCHA experience with digital devices was supported by women’s recommendations for further external environmental improvements to increase an audio-visual clarity and decrease distractions such as covering natural lights around video viewing areas and providing space with less clinic noise. CCHAs at intervention sites had successfully played the video on projectors or laptops, but at times required assistance from research staff. There were two technological challenges that occurred during the observation intervention clinics that caused delay of intervention delivery, which were power outages and the CCHA’s lack of technique in handling intervention equipment. Since power outage is a generalizable barrier in low-resource settings, we suggest future investigators to acquire additional electronic sources or backup devices for video screenings.

Although additional technological training and support were provided before the intervention, it was not sufficient for all CCHAs to play the video without assistance, nor handle power outages as these challenges delayed intervention delivery. More substantive CCHA trainings on technology management could help streamline workflow and give CCHAs confidence to troubleshoot without researchers’ supervision. Also, as a general implementation strategy, applying technology that enables video viewing both inside and outside is recommended to expand the viewing population. Therefore, it is crucial to acknowledge that implementing video-based intervention is context-specific, which requires researchers to grasp understanding of the adaptable intervention format in each setting. More substantive CCHA trainings on technology management could help streamline workflow and improve the quality of the intervention allowing CCHAs to oversee the video administration and handle the technological challenges along with the HPV self-sampling process.

We found that CCHA availability, participation, and task-sharing varied across control and intervention sites. The trend of less health talks and women screened at the intervention clinic with one designated CCHA compared to others with two CCHAs could be due to the complexity of intervention, as it required CCHAs to recruit women to listen to health talks, administer the video, and oversee HPV self-sampling and data input. This suggests that the tasks are better shared between two CCHAs rather than being solely the responsibility of one, whether it be in an intervention clinic or control clinic. The observation data consistently showed more education cycles taking place at clinics with two CCHAs present than one. Despite the differences in the number of CCHAs facilitating interventions across clinics, their abilities to handle different tasks influenced the women’s overall level of participation in the education and screening uptake.

This study evaluated a novel, locally developed video-assisted health education intervention implemented in the real-world setting. There are some limitations in the study design, including the short duration and the limited number of observations. There may have also been an influence of research staff presence on the quality and frequency of health education cycles. Researchers were not present for all education and screening cycles, so there is no direct knowledge as to whether the intervention continued as observed when researchers were not present. Although our findings showed the intervention’s promising impact, they also highlighted the need for robust structural and technologic support for intervention sustainability if the video were to be implemented as part of an HPV-based screening strategy. While our team saw a difference between the women reached with the education and screening among the intervention clinics and control clinics, this study was not powered to detect a difference, so the impact will need to be evaluated in an adequately powered study. Additionally, there is potential bias of the interest and acceptability of the video from the women who participated in FGDs, as they were selected from those who elected to watch the video, indicating their interest. Future studies may choose to conduct FGDs with control populations and women who declined participation in video education to explore suggestions to increase intervention interest. Finally, as this was a relatively small sample in a specific geographic and cultural context, the acceptability of the video may not be generalizable to other settings. Implementation processes, along with video format and content, should be rigorously tested prior to introduction in settings with different target populations.

Overall, the implementation of an HPV video education at intervention clinics was feasible and acceptable, despite technological challenges. Intervention participants expressed approval of the video education, specifically for its content and execution, speaking to the viability of long-term implementation. Although the video-supported education around HPV lasted longer, women were overall satisfied with the length, and it did not negatively impact their ability to stay and undergo screening. Finally, there were higher rates of women attending the video education and undergoing screening in the intervention group, although the study was not powered to determine a difference. There is a clear need for improved awareness, education and promotion of health behaviors around HPV, and this research demonstrated the feasibility of a video-based educational intervention as a potential solution. This study suggests that additional work to address implementation challenges will be important to ensure that this video-based intervention can be consistently offered, and the trend of a positive impact should be confirmed in an adequately powered study.

## Supporting information

S1 Fig Comparison of patient engagement in control clinics. The first two bar charts show a median number of women who received standard MoH health talks per education cycle and per day across three control clinics. The third bar chart shows a median number of women who received HPV self-sampling after the CCHA(s) delivered health talks. Control 1 clinic was observed 6 times, and control 2 and 3 clinics are observed 5 times respectively.
(TIFF)

S2 Fig Comparison of patient engagement in intervention clinics. The first two bar charts show a median number of women who received CCHA-led video-assisted HPV education per education cycle and per day across three intervention clinics. The third bar chart shows a median number of women who received HPV self-sampling after the health education. Intervention 1 and 3 clinics were observed 6 times respectively, and intervention 2 was observed 5 times.
(TIFF)

S3 Fig Comparison between control and intervention clinics. The first two bar charts show a median number of women who received CCHA-led health education across control and intervention clinics per education cycle and per day. The third bar chart shows a median number of women who received HPV self-sampling after health education delivery.
(TIFF)

S1 Table List of survey instruments for REDCap survey. (DOCX)

S1 Text Inclusivity in global research questionnaire. This questionnaire improves transparency in research performed outside of the researchers’ own country with an outline of the ethical, cultural, and scientific considerations specific to this study.
(DOCX)
