# Correction: Evaluation of video-assisted HPV education in government-supported clinics in Western Kenya

**DOI:** 10.1371/journal.pgph.0004154

**Published:** 2024-12-31

**Authors:** The PLOS GLOBAL PUBLIC HEALTH Staff

The Data Availability statement for this paper is incorrect. The correct statement is: All data for the study are deposited in the Qualitative Data Repository. The citation is: Dion, Haley; Choi, Hanul; Huang, Michelle; Sathyan, Laya; Makhulo, Breandan; Huchko, Megan. 2023. "Data for: Evaluation of Video-Assisted HPV Education in Government-Supported Clinics in Western Kenya". Qualitative Data Repository. https://doi.org/10.5064/F6ING0AD. QDR MainCollection. V1.

The publisher apologizes for the errors.
