# Supplementary figures and images for: Evaluation of video-assisted HPV education in government-supported clinics in Western Kenya
Source: PLOS Glob Public Health. 2023 Dec 18;3(12):e0002539. doi: 10.1371/journal.pgph.0002539 (PMC10727431; doi:10.1371/journal.pgph.0002539)

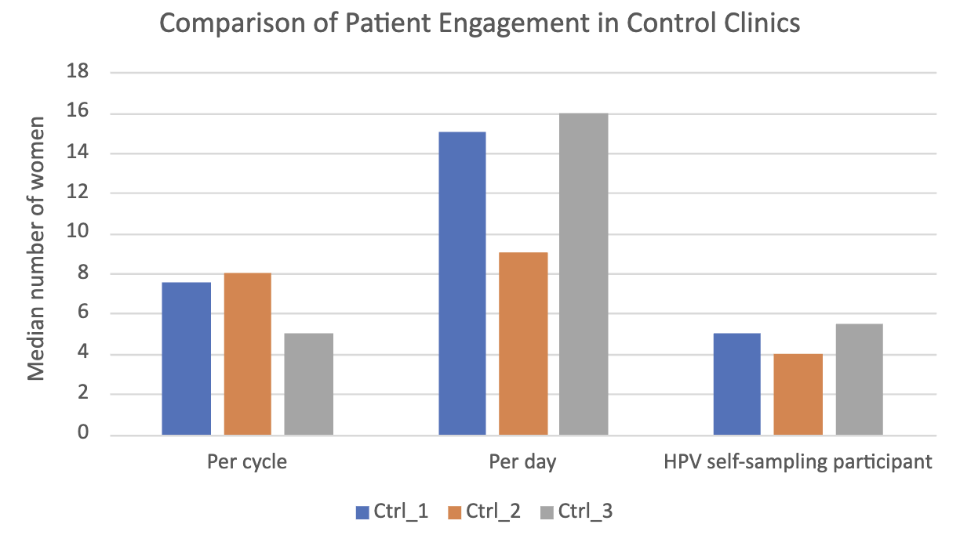

Supplement: S1 Fig — The first two bar charts show a median number of women who received standard MoH health talks per education cycle and per day across three control clinics. The third bar chart shows a median number of women who received HPV self-sampling after the CCHA(s) delivered health talks. Control 1 clinic was observed 6 times, and control 2 and 3 clinics are observed 5 times respectively. (TIFF) [file pgph.0002539.s001.tiff]

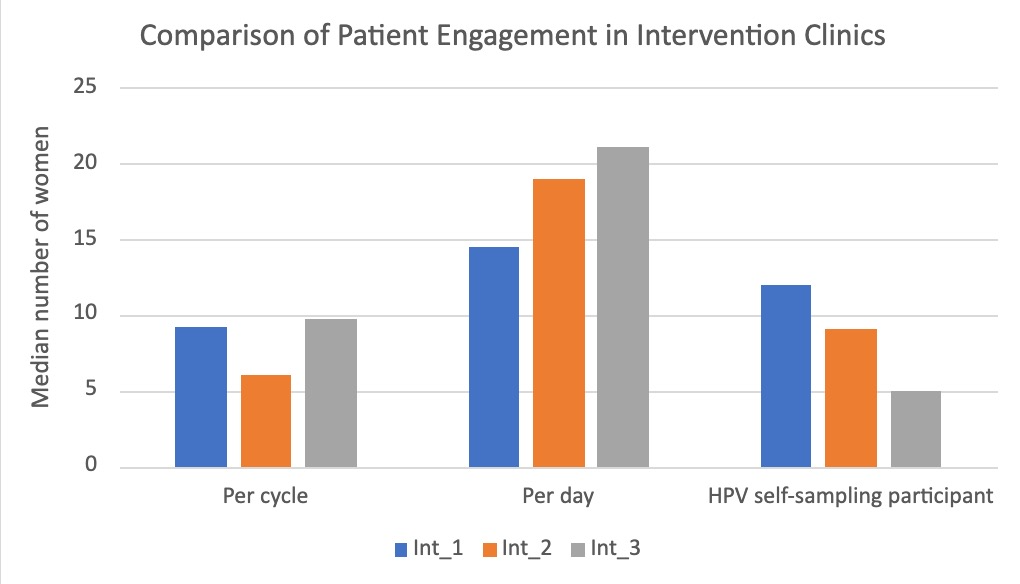

Supplement: S2 Fig — The first two bar charts show a median number of women who received CCHA-led video-assisted HPV education per education cycle and per day across three intervention clinics. The third bar chart shows a median number of women who received HPV self-sampling after the health education. Intervention 1 and 3 clinics were observed 6 times respectively, and intervention 2 was observed 5 times. (TIFF) [file pgph.0002539.s002.tiff]

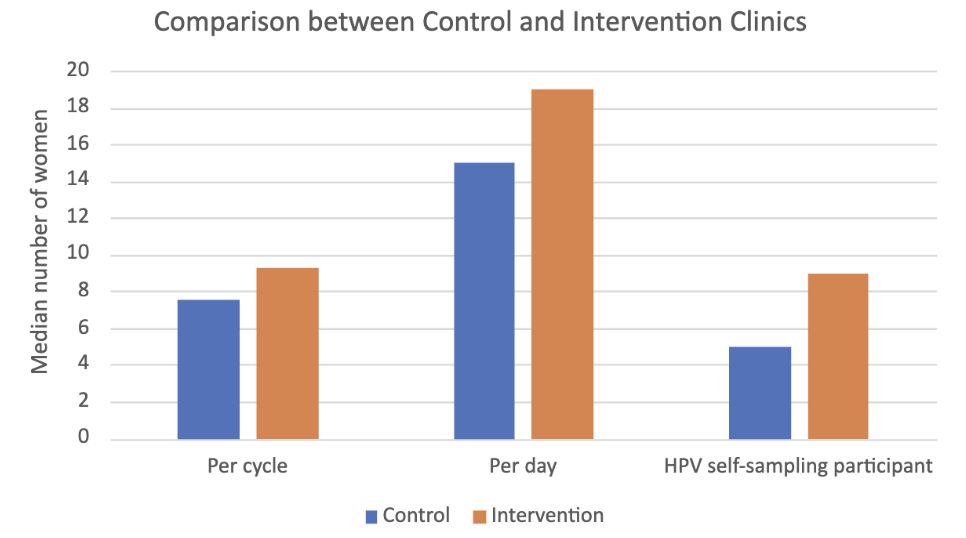

Supplement: S3 Fig — The first two bar charts show a median number of women who received CCHA-led health education across control and intervention clinics per education cycle and per day. The third bar chart shows a median number of women who received HPV self-sampling after health education delivery. (TIFF) [file pgph.0002539.s003.tiff]
