# Supplementary material for: Evaluation of video-assisted HPV education in government-supported clinics in Western Kenya
Source: PLOS Glob Public Health. 2023 Dec 18;3(12):e0002539. doi: 10.1371/journal.pgph.0002539 (PMC10727431; doi:10.1371/journal.pgph.0002539)
Supplement: S1 Table — (DOCX) [file pgph.0002539.s004.docx]

S1 Table: List of survey instruments for REDCap survey

| Variable / Field Name | Form Name | Field Type | Field Label | Choices, Calculations, OR Slider Labels |
| --- | --- | --- | --- | --- |
| record_id | Clinic information | text | Record ID |  |
| observer_initials | Clinic information | text | Observer Initials |  |
| date | Clinic information | text | Date |  |
| clinic_name | Clinic information | radio | Clinic Name | 1, Clinic 1 \| 2, Clinic 2\| 3, Clinic 3\| 4, Clinic 4\| 5, Clinic 5\| 6, Clinic 6 |
| control_or_intervention | Clinic information | radio | Control or Intervention | 1, Control \| 2, Intervention |
| observation_start_time | Clinic information | text | Observation Start Time (24hrs) |  |
| observation_end_time_24hrs | Clinic information | text | Observation End Time (24hrs) |  |
| control_clinic | Control Clinic | yesno | Is this control clinic? |  |
| waiting_time | Control Clinic | text | How long were women generally waiting to see providers? |  |
| are_there_CCHAs | Control Clinic | yesno | Were there CCHAs delivering (HPV and cervical cancer) health education? |  |
| education_frequency | Control Clinic | text | How many times was the educational health information provided during the prior week? |  |
| education_weekly | Control Clinic | checkbox | What days was it provided? | 1, Monday \| 2, Tuesday \| 3, Wednesday \| 4, Thursday \| 5, Friday |
| education_daily | Control Clinic | text | How many times is the HPV and cervical cancer educational health information provided per day? |  |
| education_during_observation | Control Clinic | yesno | Was there HPV and cervical cancer health education offered during the observation? |  |
| education_length | Control Clinic | text | How long were the health education cycles? |  |
| women_talking_CCHA | Control Clinic | text | How many women are present at once for the health education cycle? |  |
| total_women | Control Clinic | text | How many women total women attended health education cycles today? |  |
| education_interruption | Control Clinic | yesno | Were there any interruptions in delivering educational health information? |  |
| interruption_frequency | Control Clinic | text | How many times? |  |
| interruption_details | Control Clinic | text | Please elaborate on the interruptions. |  |
| designated_workers | Control Clinic | yesno | Besides CCHAs, are there designated workers providing the educational health information? |  |
| who_are_workers | Control Clinic | checkbox | Who are they? | 1, Clinic staff (non-medical) \| 3, Nurse (including PAs, LPNs) \| 4, Provider \| 5, Other |
| workers_specify | Control Clinic | text | Please specify: |  |
| how_many_workers | Control Clinic | text | How many designated workers were there delivering educational health information? |  |
| women_with_dependents | Control Clinic | text | How many women visited the clinic with dependents (e.g. children, spouse, etc.)? |  |
| women_dependents_educ | Control Clinic | text | How many women were listening to the health information session with their dependents? |  |
| women_with_dependents_hpv | Control Clinic | text | How many women with dependents received HPV self-sampling? |  |
| how_many_screened | Control Clinic | text | How many women got screened for HPV during the observation? |  |
| stockouts_yes_no_ctrl | Control Clinic | yesno | In the last week, were there any stockouts of HPV test kits? |  |
| hpv_testkit_stockout | Control Clinic | checkbox | In the last week, on which days were there stockouts of HPV test kits? | 1, Monday \| 2, Tuesday \| 3, Wednesday \| 4, Thursday \| 5, Friday |
| additional_services | Control Clinic | yesno | Are there any other HPV related services offered in this clinic on different days? |  |
| add_services_specify | Control Clinic | notes | Please specify: |  |
| additional_information | Control Clinic | notes | Additional Information to note: |  |
| Intervention Clinic | Intervention Clinic | yesno | Is this intervention clinic? |  |
| waiting_time_2 | Intervention Clinic | text | How long were women generally waiting to see providers? |  |
| video_shown | Intervention Clinic | yesno | Were educational videos shown to patients? |  |
| education_frequency_2 | Intervention Clinic | text | How many times was the educational health information provided during the prior week? |  |
| education_weekly_2 | Intervention Clinic | checkbox | What days was it provided? | 1, Monday \| 2, Tuesday \| 3, Wednesday \| 4, Thursday \| 5, Friday |
| video_frequency | Intervention Clinic | text | How many times was the video shown during the day? |  |
| women_watching_video | Intervention Clinic | text | How many women watch the video at once? |  |
| total_women_video | Intervention Clinic | text | How many total women watched the video today? |  |
| education_cycle_length | Intervention Clinic | text | How long was each video/ health education cycle? |  |
| video_showing_description | Intervention Clinic | notes | Describe how the video was shown in the clinic. (location, method, person in charge) |  |
| other_videos | Intervention Clinic | yesno | Is the video alternated with other health education topics? |  |
| video_staff_members | Intervention Clinic | yesno | Are there any clinic staff members who oversee the video play? |  |
| video_staff_members_2 | Intervention Clinic | notes | Who are they and what are their other duties? |  |
| video_interruption | Intervention Clinic | yesno | Were there any video interruptions during the observation? |  |
| video_interrupt_frequency | Intervention Clinic | text | How many times? |  |
| reason_video_interruption | Intervention Clinic | yesno | Were there any power outages? |  |
| reason_video_interruption_2 | Intervention Clinic | yesno | Did the equipment break? |  |
| reason_video_interruption_3 | Intervention Clinic | notes | What were other reasons of interruptions? |  |
| designated_workers_2 | Intervention Clinic | yesno | Are there designated workers providing the educational health information? |  |
| who_are_workers_2 | Intervention Clinic | checkbox | Who are they? | 1, Clinic staff (non-medical) \| 2, Community health volunteer (CCHA) \| 3, Nurse (including PAs, LPNs) \| 4, Provider \| 5, Other |
| workers_specify_2 | Intervention Clinic | text | Please specify: |  |
| how_many_workers_2 | Intervention Clinic | text | How many? |  |
| additional_activities | Intervention Clinic | yesno | Are any activities conducted with the patient before rooming/watching videos? |  |
| additional_activities_2 | Intervention Clinic | notes | Please specify: |  |
| additional_activities_3 | Intervention Clinic | checkbox | Who conducts these activities? | 1, Clinic staff (non-medical) \| 2, Community health volunteer (CCHA) \| 3, Nurse (including PAs, LPNs) \| 4, Provider \| 5, Other |
| additional_activities_4 | Intervention Clinic | notes | Please specify: |  |
| women_with_dependents_2 | Intervention Clinic | text | How many women visited the clinic with dependents (e.g. children, spouse, etc.)? |  |
| women_dependents_video | Intervention Clinic | text | How many women were watching the educational video with their dependents? |  |
| women_with_dependents_hpv_2 | Intervention Clinic | text | How many women with dependents received HPV self-testing? |  |
| how_many_women_received_hp | Intervention Clinic | text | How many women received HPV self-testing? |  |
| stockouts_yes_no | Intervention Clinic | yesno | In the last week, were there any stockouts of HPV testing kits? |  |
| hpv_testkit_stockout_3 | Intervention Clinic | checkbox | In the last week, on which days were there stockouts of HPV test kits? | 1, Monday \| 2, Tuesday \| 3, Wednesday \| 4, Thursday \| 5, Friday |
| additional_services_2 | Intervention Clinic | yesno | Are there any other HPV related services offered in this clinic on different days? |  |
| add_services_3 | Intervention Clinic | notes | Please specify: |  |
| additional_information_2 | Intervention Clinic | notes | Additional information to note: |  |
